# Long COVID following SARS‐CoV‐2 infection during pregnancy: An observational study in a large Italian hospital during the COVID‐19 pandemic

**DOI:** 10.1111/aogs.70127

**Published:** 2026-02-13

**Authors:** Anna Fichera, Eleonora Biancareddu, Marco Bozzo, Mirabella Ezenwa, Emma Paola Ongarini, Federico Giorgio Ferrari, Federico Prefumo, Franco Edoardo Odicino

**Affiliations:** ^1^ Department of Clinical and Experimental Sciences University of Brescia Brescia Italy; ^2^ Obstetrics and Gynecology Unit ASST‐Spedali Civili Brescia Italy; ^3^ Obstetrics and Gynecology Unit IRCCS Istituto Giannina Gaslini Genova Italy

**Keywords:** long COVID, pandemic, PASC, pregnancy, SARS‐CoV‐2 infection

## Abstract

**Introduction:**

Despite mounting evidence on Long COVID, data regarding its impact on women infected during pregnancy remains scarce. This study aimed to assess the development of Long COVID in women who had been infected with SARS‐CoV‐2 during pregnancy, focusing on possible risk factors and potential protective elements associated with its development.

**Material and Methods:**

We analyzed a cohort of 348 pregnant women with laboratory‐confirmed SARS‐CoV‐2 infection admitted to ASST‐Spedali Civili (Brescia, Italy) between March 2020 and May 2022. Data collection included demographics, comorbidities, COVID‐19 severity markers, and vaccination status. To assess the possible association between the analyzed risk factors and Long Covid, beyond standard multivariable models, we employed inverse probability weighting techniques (IPTW) and calculated e‐values to assess unmeasured confounding.

**Results:**

Among study participants, 27.0% (94/348) developed Long COVID. Risk factors included preexisting respiratory comorbidities (adjusted OR = 3.171, 95% CI 0.99–10.1), pneumonia at admission (adjusted OR = 4.48, 95% CI 2.16–9.28), and earlier pregnancy stage at infection (adjusted OR = 0.96 per week, 95% CI 0.93–0.99). COVID‐19 vaccination was associated with a significantly lower risk of Long COVID (15.5% in vaccinated vs. 31.8% in unvaccinated women; IPTW‐adjusted OR = 0.38, 95% CI: 0.20–0.71, p‐value: 0.003). The most common symptoms were fatigue (46.8%) and memory impairment (38.3%), with unvaccinated patients exhibiting a higher prevalence of neuropsychiatric symptoms.

**Conclusions:**

Our data suggest that one in four pregnant women hospitalized with COVID‐19 develop persistent symptoms. The most frequently affected women had preexisting respiratory disease, pneumonia at admission, and infection earlier in pregnancy. COVID‐19 vaccination appears to reduce risk and alter symptom presentation. These findings underscore the importance of vaccination throughout pregnancy and highlight the need for targeted surveillance in high‐risk subgroups.

## Graphical abstract

Among 348 women infected with SARS‐CoV‐2 during pregnancy, 27% developed Long COVID. Risk factors included preexisting respiratory comorbidities, pneumonia, and earlier pregnancy stage. Vaccination significantly reduced this risk and altered symptom patterns, supporting its protective role and emphasizing the importance of preventive strategies for pregnant populations at higher risk.

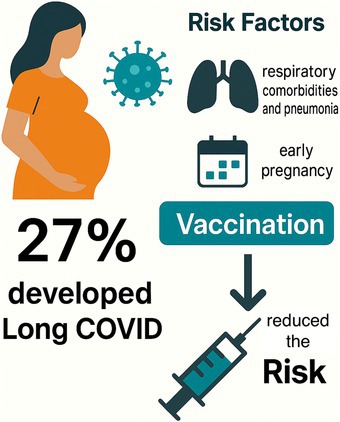

**Key message**

One in four women infected with SARS‐CoV‐2 during pregnancy developed Long COVID. Vaccination significantly reduced this risk. Our findings highlight the need for targeted monitoring and prevention strategies in vulnerable pregnant populations.

## 1 INTRODUCTION

SARS‐CoV‐2 infection has been associated with some unique risks during pregnancy. Pregnant women are more likely to experience severe illness compared to the nonpregnant population and may require more intensive life‐supporting measures, admission to the intensive care unit (ICU), mechanical ventilation, or ECMO (extracorporeal membrane oxygenation).^1^ Moreover, SARS‐CoV‐2 infection during pregnancy has also been linked to several pregnancy‐specific complications, like preterm birth and stillbirth.^2^, ^3^ This increased vulnerability has been hypothesized to be related, among other changes, to the pregnancy‐specific immune modifications, such as a shift from Th1 to Th2 immune responses and reduced Natural Killer (NK) cell activity, that may alter viral clearance mechanisms.^4^, ^5^ The postacute sequelae of SARS‐CoV‐2 (PASC), or Long COVID, are now a recognized consequence of the pandemic, affecting potentially 10–30% of infected individuals. This syndrome involves symptoms that might not be present before acute illness and that linger or appear a few months after what should have been a resolved case of COVID‐19.^6^ With its status as a newly recognized condition, the World Health Organization has defined Long COVID as symptoms occurring within 3 months of SARS‐CoV‐2 infection, persisting for at least 2 months, and not explained by alternative diagnoses.^7^ Common manifestations of Long COVID include fatigue, shortness of breath, and cognitive dysfunction.^8^ These symptoms appear to affect women approximately 13% more often than men.^6^ In the pregnant population, prevalence rates range widely from as low as 9.3% up to 74.7%. This high variability is probably partly dependent on the definition of Long COVID the authors employed as well as different inclusion criteria and information‐gathering protocols^3^, ^9^, ^10^

COVID‐19 vaccination during pregnancy has demonstrated protective effects against adverse outcomes.^11^, ^12^ Recent meta‐analyses show that vaccination reduces the risk of SARS‐CoV‐2 infection (by ~60%), hospitalization (~53%), and ICU admission (~82%) in pregnant women.^13^ Additionally, vaccination has been linked to a reduced risk of preterm birth and stillbirth^1^

Nonetheless, while systematic reviews suggest vaccination decreases Long COVID risk by about 24% in general populations,^14^ data specific to pregnant women remain scarce. Our study aims to evaluate the development of Long COVID in pregnant women following SARS‐CoV‐2 infection, characterizing the symptom profile and identifying potential risk or protective factors within this specific population.

## 2 MATERIAL AND METHODS

This observational study combined retrospective data collection from medical records with prospective symptom assessment via questionnaires. It was conducted at the Obstetrics and Gynecology Unit of ASST‐Spedali Civili of Brescia (Italy), a tertiary care center that served as a regional reference hub for COVID‐19 pregnancies from March 2020 to May 2022. Informed consent was obtained from all participants prior to their inclusion in the study.

The study included pregnant women aged ≥18 years with confirmed SARS‐CoV‐2 infection via reverse transcription polymerase chain reaction (RT‐PCR) or antigen testing, either during pregnancy at any gestational age or upon admission for labor. Long COVID was defined as persistence of symptoms or emergence of new ones for more than 12 weeks following acute infection. Data were primarily extracted from electronic medical records using institutional hospital digital systems. Information collected included demographic data (age, body mass index (BMI), country of origin), maternal comorbidities (obesity, diabetes, hypertension, cardiovascular problems, history of respiratory disease, autoimmune conditions), COVID‐19 infection details (date of admission, severity, hospitalization duration, ICU admission, pneumonia diagnosis), pregnancy‐related data (gestational age at infection, obstetric complications, mode of delivery and pregnancy outcome).

A standardized questionnaire detailed in Table S1 was specifically developed for this study and administered to eligible patients through telephone interviews. We asked about symptoms characteristics, duration, severity, and effect on quality of life, other than vaccination status at the moment of admission. The list of symptoms was developed based on the most frequently described manifestations in the literature on post‐acute COVID‐19 sequelae, as reported in major clinical studies, while at the same time allowing participants to specify which symptoms they experienced. This approach allowed participants to report additional manifestations beyond the predefined list. Based on questionnaire responses, participants were divided into two groups: those who experienced only acute symptoms and those who developed long‐term sequelae persisting beyond 12 weeks.

### 2.1 Statistical analysis

Descriptive statistics (means, standard deviations, and proportions) were calculated for the total cohort and stratified by Long COVID status. Continuous variables were compared using Student's t‐test, and categorical variables using chi‐squared or Fisher's exact test as appropriate.

To identify Long COVID risk and protective factors within the overall cohort, we constructed a multivariable logistic regression model. Covariates were selected for inclusion based on a combination of clinical relevance, biological plausibility, and statistical criteria (univariate *p* < 0.1) to ensure model parsimony, avoid overfitting given the event rate, and maintain >10 events per variable.^15^, ^16^ The final model adjusted for potential confounders: age, BMI, preexisting respiratory disease, pneumonia at admission, ICU admission, vaccination status, gestational age at infection, diabetes, and cardiovascular risk factors. Other variables captured in the questionnaire (Table S1), such as smoking, were excluded due to nonsignificant univariate associations and low prevalence, which could risk model instability. Results are presented as adjusted odds ratios (aOR) with 95% confidence intervals. Model fit was confirmed by the Hosmer‐Lemeshow test.

Finally, to robustly assess vaccination effects while addressing potential confounding, propensity scores were created for application in inverse probability of treatment weighting (IPTW). For this specific IPTW analysis, we excluded women for whom the infection was diagnosed at the time of admission for delivery, as gestational age at COVID‐19 infection during pregnancy—a key covariate in the propensity score model—was not applicable for them. Covariates were selected based on clinical reasoning and established literature on confounders in pregnancy‐related COVID‐19 outcomes, including factors influencing both vaccination likelihood (e.g., per Italian guidelines prioritizing comorbidities during 2020–2022) and Long COVID risk (e.g., respiratory issues).^17^, ^18^

The propensity score model for IPTW included age, BMI, pre‐existing comorbidities (respiratory disease, diabetes, hypertension, cardiac disease, autoimmune disorders), and gestational age at infection. Variables like smoking, captured via the questionnaire (Table S1), were excluded due to low prevalence and potential noise in our cohort. Balance after weighting was assessed using standardized mean differences with values. Balance after weighting was assessed using standardized mean differences, with values <0.1 considered adequate.

The propensity scores were estimated using a logistic regression model via the glm() function from the base stats package in R. Stabilized IPTW weights (swi) were then manually calculated for each subject using the following formula to improve stability and reduce variance:

For vaccinated subjects (Ti = 1):

$$\mathrm{swi}=\frac{P\left(T=1\right)}{e\left(\mathrm{xi}\right)}$$

and for unvaccinated subjects (Ti = 0)

$$\mathrm{swi}=\frac{P\left(T=0\right)}{1-e\left(\mathrm{xi}\right)},$$

where *P*(*T* = 1) was the overall proportion of vaccinated subjects in this analytical sample, *P*(*T* = 0) was the proportion of unvaccinated subjects, and *e*(*xi*) was the propensity score for subject *i*. These stabilized weights were then trimmed at the first and 99th percentiles. Standardized mean differences (SMDs) were calculated for all covariates using custom R functions based on standard formulae. An SMD <0.1 was considered to indicate a negligible imbalance between groups after weighting.

Associations between vaccination status (and other covariates) and Long COVID in the IPTW‐weighted cohort were estimated using univariable and multivariable logistic regression models. These weighted logistic regressions were performed using the svyglm() function from the survey package in R. Results from these weighted models are presented as odds ratios (OR) with 95% confidence intervals.

To evaluate robustness to unmeasured confounding, E‐values for key associations were calculated. The E‐value represents the minimum strength of association that an unmeasured confounder would need to have with both exposure and outcome to nullify the observed relationship.

All analyses were performed using SPSS Statistics 26 (SPSS Inc., Chicago, USA) and R version 4.4.3 (R Foundation for Statistical Computing, Vienna, Austria). A two‐sided p‐value <0.05 was considered statistically significant.

## 3 RESULTS

**FIGURE 1:**
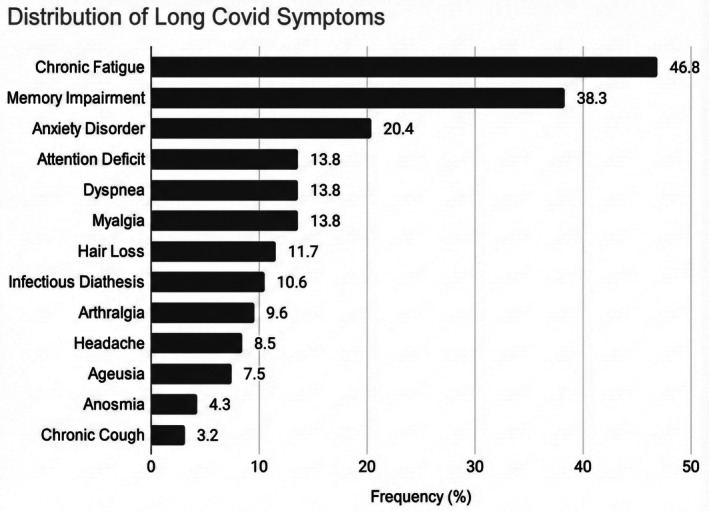
Overall distribution of Long COVID symptoms (*n* = 94).

**FIGURE 2:**
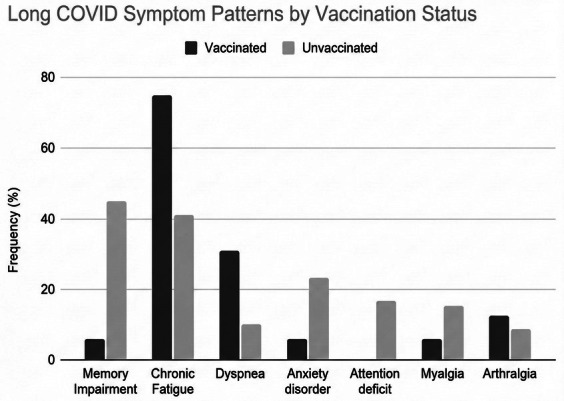
Grouped bar chart comparing symptom frequencies between vaccinated and unvaccinated Long COVID patients.

A total of 510 women were identified. Of these, 133 could not be reached and 29 declined to participate. In the end, 348 pregnant women, hospitalized with COVID‐19 across all levels of severity, were included in the study (68.2%): 103 (30%) were vaccinated and 245 (70%) were unvaccinated. Among them, 94 (27.0%) developed Long COVID: the most frequently reported persistent symptoms were chronic fatigue (46.8%), memory impairment (38.3%), anxiety disorders (20.4%), attention deficit, dyspnea, and myalgia (each 13.8%) (Figure 1). The mean number of symptoms reported per patient was 1.9 (range 1–7), with 63.8% of patients reporting multiple persistent symptoms. Symptom patterns differed markedly by vaccination status (Figure 2). Among vaccinated patients with Long COVID (*n* = 16), chronic fatigue was most common (75.0%), followed by dyspnea (31.2%) and arthralgia (12.5%). In contrast, unvaccinated patients (*n* = 78) exhibited a broader symptom profile, with memory impairment (44.9%), chronic fatigue (41.0%), and anxiety disorders (23.4%) being most common.

Baseline characteristics of the study population stratified by Long COVID status are reported in Table 1 as well as univariate logistic regression analysis. Compared to those without Long COVID (*n* = 254), affected women had significantly longer hospital stays (mean 7.6 ± 7.5 vs. 4.6 ± 3.7 days, *p* = 0.002), higher rates of ICU admission (6.4% vs. 1.2%, *p* = 0.025), were more likely to have radiologically confirmed pneumonia (35.1% vs. 8.3%, *p* < 0.001) and a history of pre‐existing respiratory disease (9.6% vs. 2.4%, *p* = 0.013). Notably, patients who developed Long COVID were infected at an earlier gestational age compared to those who did not (mean 33.3 ± 8.4 vs. 36.2 ± 6.4 weeks, *p* = 0.001). Vaccination rates were also markedly lower in the Long COVID group (17.0% vs. 34.3%, *p* = 0.002). No significant differences were observed between groups regarding other comorbidities, including diabetes, chronic hypertension, cardiac disease, and autoimmune or endocrine disorders. Women with respiratory disease had significantly higher rates of Long COVID compared to those without (60.0% vs. 25.5%, *p* = 0.013).

**TABLE 1:** Baseline characteristics of the study population according to Long COVID status.

Characteristic	Long COVID	No Long COVID	*p*‐value
Demographics			
*N*	94	254	—
Age, years, mean ± SD	33.1 ± 6	32.1 ± 5.5	0.338
BMI, kg/m^2^, mean ± SD	28.2 ± 6	27.8 ± 5.8	0.732
Clinical characteristics			
Vaccinated	16 (17.0)	87 (34.3)	0.002
ICU admission	6 (6.4)	3 (1.2)	0.014
Length of stay, days, mean ± SD	7.6 ± 7.5	4.6 ± 3.7	0.002
Pneumonia at admission	26 (27.7)	16 (6.3)	<0.001
Gestational age at admission, weeks, mean ± SD	33.3 ± 8.4	36.2. ± 6.4	0.001
Comorbidities			
Obesity (BMI ≥ 30)	25 (26.6)	57 (22.4)	0.280
Diabetes	1 (1.1)	5 (2.0)	0.847
Chronic hypertension	2 (2.1)	4 (1.6)	0.940
Respiratory disease	9 (9.6)	6 (2.4)	0.006
Cardiac disease	3 (3.2)	2 (0.8)	0.247
Autoimmune disease	2 (2.1)	8 (3.1)	0.880
Endocrine disease	8 (8.5)	17 (6.7)	0.844

*Note*: Values are presented as *n* (%) unless otherwise specifies. P‐values are calculated using Student's *t*‐test for continuous variables and chi‐squared or Fisher's exact test for categorical variables.

Abbreviations: BMI, body mass index; ICU, Intensive Care Unit; SD, standard deviation.

In the multivariable logistic regression model, after adjustment for potential confounders, including age, BMI, pneumonia at admission, ICU admission, cardiovascular risk factors, gestational age at admission, diabetes, and vaccination status, preexisting respiratory disease remained independently associated with Long COVID development (adjusted OR = 3.17, 95% CI 1.21–12.39, *p* = 0.045; E‐value = 5.8).

Pneumonia at admission was the strongest predictor of Long COVID in our cohort. Women diagnosed with pneumonia at admission had significantly higher rates of Long COVID compared to those without pneumonia (61.9% vs. 21.2%, *p* < 0.001). After multivariable analysis, pneumonia at admission remained strongly associated with Long COVID development (adjusted OR = 4.481, 95% CI: 2.16–9.28, p < 0.001; E‐value 9.51).

Gestational age at SARS‐CoV‐2 infection emerged as a significant independent predictor of Long COVID development: each additional week of pregnancy at the time of infection was associated with a 3.9% reduction in the odds of developing Long COVID (adjusted OR = 0.961 per week, 95% CI: 0.928–0.995, *p* = 0.027). This inverse relationship remained significant after adjusting for maternal age, BMI, preexisting respiratory disease, pneumonia at admission, vaccination status, diabetes, and cardiovascular risk factors.

Vaccination status significantly influenced Long COVID risk. In unadjusted analysis, 15.5% of vaccinated patients developed Long COVID versus 31.8% of unvaccinated patients (*p* = 0.002; risk ratio = 0.394). After adjusting for relevant covariates, including disease severity indicators, the association was attenuated and no longer statistically significant (adjusted OR = 0.60, 95% CI: 0.31–1.16, *p* = 0.13), although the direction of effect remained consistent.

To better estimate the overall impact of vaccination, including indirect effects via reduced disease severity, an inverse probability of treatment weighting (IPTW) analysis was conducted on a sub‐cohort of 326 women (89 vaccinated, 237 unvaccinated). This sample excluded women for whom the infection was diagnosed at the time of admission for delivery, for whom gestational age at infection was not applicable for propensity score modeling. The estimated propensity scores in this sample ranged from 0.101 to 0.508, and the trimmed stabilized weights ranged from 0.694 to 1.703. Baseline characteristics of this analytical sample (*N* = 326) before and after IPTW weighting are presented in Table 2. After applying the trimmed stabilized IPTW, we achieved a balance across all measured covariates included in the propensity score model, with postweighting SMDs below 0.1 (e.g., for age, postweighting SMD = 0.011; for BMI, postweighting SMD = 0.041).

**TABLE 2:** Baseline characteristics of the IPTW analytical cohort (*N* = 326) before and after inverse probability of treatment weighting.

Characteristic	Vaccinated (*N* = 89)	Unvaccinated (*N* = 237)	SMD (pre‐Wt.)	Vaccinated (weighted)	Unvaccinated (weighted)	SMD (post‐Wt.)
Age (years), mean (SD)	33.2 (±5.1)	32.1 (±5.8)	0.204	32.5 (±5.3)	32.5 (±5.8)	0.011
BMI (kg/m^2^), mean (SD)	26.2 (±6.0)	27.4 (±5.0)	−0.222	27.4 (±6.7)	27.1 (±4.9)	0.041
Gestational age at admission (weeks), mean (SD)	35.3 (±7.0)	35.5 (±7.2)	−0.032	35.1 (±7.4)	35.4 (±7.4)	−0.047
Preexisting comorbidities, *N* (%)						
Respiratory disease	3 (3.4%)	12 (5.1%)	−0.084	3 (3.8%)^a^	12 (4.6%)^a^	−0.036
Diabetes	1 (1.1%)	5 (2.1%)	−0.078	1 (1.4%)^a^	5 (1.8%)^a^	−0.032
Chronic hypertension	1 (1.1%)	5 (2.1%)	−0.078	1 (1.0%)^a^	5 (1.8%)^a^	−0.062
Cardiac disease	2 (2.2%)	3 (1.3%)	0.075	2 (1.7%)^a^	3 (1.6%)^a^	0.006
Autoimmune disease	2 (2.2%)	8 (3.4%)	−0.068	2 (2.6%)^a^	8 (3.0%)^a^	−0.025

*Note*: Values for continuous variables are mean (SD); values for categorical variables are *N* (%). SMDs compare vaccinated vs. unvaccinated groups. Abbreviations: SMD, standardized mean difference; Wt., weighting.

^a^ Counts and percentages for postweighting categorical variables reflect the weighted distribution based on re‐analysis (*N* = 326: vaccinated, *N* = 89, unvaccinated, *N* = 237).

The results from the IPTW‐weighted logistic regression analyses for Long COVID, performed on the N = 326 cohort, are shown in Table 3. This approach yielded a weighted odds ratio of 0.38 (95% CI: 0.20–0.71, *p* = 0.003), indicating a statistically significant 62% reduction in the odds of Long COVID among vaccinated women. The corresponding E‐value of 4.70 (2.17 for the lower CI bound) indicated that a relatively strong unmeasured confounder would be needed to nullify this effect, supporting a potentially causal relationship between vaccination and reduced Long COVID risk.

**TABLE 3:** Association between vaccination, baseline covariates, and Long COVID from IPTW‐weighted logistic regression analyses.

Variable	Univariate OR (95% CI)	*p*‐value	Multivariate OR (95% CI)^a^	*p*‐value
Vaccination (yes vs. no)	0.44 (0.23–0.82)	0.011	0.38 (0.20–0.71)	0.003
Age (per year)	1.03 (0.99–1.08)	0.186	1.03 (0.98–1.08)	0.256
BMI (per unit kg/m^2^)	1.04 (1.00–1.09)	0.061	1.06 (1.01–1.11)	0.015
Gestational age at admission (per week)	0.95 (0.92–0.98)	0.002	0.94 (0.91–0.98)	0.001
Preexisting comorbidities (yes vs. no)				
Respiratory disease	3.92 (1.34–11.48)	0.013	3.07 (1.18–8.01)	0.022
Diabetes	0.48 (0.06–4.24)	0.513	0.35 (0.03–3.89)	0.397
Chronic hypertension	1.22 (0.22–6.83)	0.817	0.66 (0.08–5.36)	0.702
Cardiac disease	4.79 (0.75–30.47)	0.098	4.04 (0.60–27.08)	0.151
Autoimmune disease	0.58 (0.12–2.79)	0.496	0.76 (0.16–3.58)	0.727

*Note*: All models are IPTW‐weighted using trimmed stabilized weights on the *N* = 326 analytical sample.

Abbreviations: CI, confidence interval; IPTW, inverse probability of treatment weighting; OR, odds ratio.

^a^ Multivariable model adjusted for vaccination status and all listed covariates simultaneously.

Regarding specific symptom differences by vaccination status in the overall cohort (*N* = 348): memory impairment was significantly less prevalent in vaccinated compared to unvaccinated patients (6.2% vs. 44.9%, *p* = 0.004). Similarly, attention deficit (0% vs. 16.7%), infectious diathesis (0% vs. 12.8%), anosmia (0% vs. 5.1%), and ageusia (0% vs. 9.0%) were entirely absent in vaccinated patients but present in varying proportions of unvaccinated patients.

## 4 DISCUSSION

Our study in women hospitalized with COVID‐19 at the regional hub of one of the most affected Italian cities by COVID‐19 found that approximately one‐quarter (27.0%) experienced Long COVID. Our analysis points to several key factors increasing this risk: underlying respiratory illness before pregnancy, developing pneumonia during the acute infection, and getting infected earlier in gestation all independently predicted Long COVID. Conversely, COVID‐19 vaccination appeared to be a protective factor, associated with both a lower overall chance of developing Long COVID and a different pattern of symptoms.

Comparing our findings directly to other recent studies can be difficult. The landscape of Long COVID research in pregnancy is characterized by different patient groups, definitions, and ways of collecting data. Our 27.0% rate falls within the broad range (9.3% to over 90%) found across different studies.^19^ It is substantially higher than the 9.3% seen in the large US RECOVER‐Pregnancy Cohort,^3^ a group that was not exclusively hospitalized and was mostly infected later in the pandemic (Omicron wave). The incidence of Long COVID in our cohort seems to be higher also than rates derived from massive US electronic health record databases using computational phenotypes, ranging from 4.4% to 16.5%.^20^ This difference likely stems from our focus on hospitalized patients—a group inherently likely to have had more severe acute illness, which is itself a major driver of Long COVID risk.^3^, ^9^, ^10^ Our rate does align more closely with the 34.2% reported by a Spanish study that included outpatients but used a similar ≥3‐month definition.^9^ It remains much lower, though, than the 74.7% reported in a single‐center Turkish study which employed a broader >4‐week persistence definition.^10^ This sheer variability probably reflects the different study design choices: inclusion criteria (hospitalized vs. all pregnant individuals), Long COVID definition (different duration), and how information was gathered (patient interviews vs. record mining). The period of investigation also seems to be relevant, with Omicron having reported substantially lower absolute incidences.^21^

A consistent finding across our study and others is the strong association between acute COVID‐19 severity and subsequent Long COVID risk. Our identification of pneumonia at admission (aOR 4.48) and ICU admission (significant in univariate analysis) aligns well with findings associating oxygen requirement,^3^ hospitalization,^9^, ^10^ or moderate/severe acute disease with increased Long COVID risk.^9^ Our finding that prior respiratory disease might also independently raise Long COVID risk (aOR 3.17) further highlights underlying vulnerability. Other studies based on the general population pointed out that preexisting asthma and COPD were associated with increased odds of developing Long COVID by 41% and 32%, respectively.^22^ Perhaps altered local immune function, reduced viral clearance capacity, or simply less physiological buffer make persistent symptoms more likely or noticeable. This is consistent with observations in non‐pregnant groups and aligns with the broader comorbidity risks observed in pregnancy cohorts.^3^, ^20^

We found that increasing gestational age has a 4% reduced odds per week of developing Long COVID. Zang et al. (2025) found higher risks with first/second‐trimester infection, while Muñoz‐Chápuli Gutiérrez et al. (2024) with first and third trimester infection.^9^, ^20^ Our result, coming from hospitalized patients, could imply that needing hospitalization due to COVID‐19 early in pregnancy might signify greater maternal susceptibility or a different interaction with the developing immune system at that stage, potentially hindering full recovery. Improving maternal antibody function may also have a role later in gestation.^23^ However, some symptoms could be confounded by overlapping pregnancy physiology (e.g., fatigue/dyspnea from metabolic demands). Mitigating this, assessments occurred postdelivery (mean 12–18 months post‐infection). More work is clearly needed to dissect how gestational timing, immune changes, viral factors, and Long COVID truly intersect.

Our data show that COVID‐19 vaccination offers protection against Long COVID in our cohort. While the effect was attenuated after adjusting for acute severity markers—suggesting preventing severe illness is a key benefit—the strong protective signal seen in unadjusted and IPTW analyses (weighted aOR 0.38) supports a meaningful overall effect, with an approximately 62% lower odds of Long COVID in vaccinated individuals. This finding aligns with other research^9^ and systematic reviews, identifying nonvaccination as a risk factor and suggesting vaccination helps reduce Long COVID.^9^, ^13^, ^14^, ^19^ The absence of a significant adjusted association in the RECOVER cohort could arise from differences in study populations (predominantly Omicron era), statistical power, or adjustment strategies.^24^

Intriguingly, our study also hints that vaccination might change *how* Long COVID presents during pregnancy. We found a marked reduction in neuropsychiatric symptoms, especially memory impairment, among vaccinated women compared to unvaccinated women (6.2% vs. 44.9%). Although fatigue remained frequent in both groups, this apparent shift away from cognitive issues in vaccinated individuals mirrors some observations in general populations.^25^

Our results identify specific high‐risk groups—particularly pregnant women with respiratory comorbidities or severe acute disease—who are at higher risk of Long COVID development.

These risk factors—respiratory comorbidities (aOR = 3.17), pneumonia (aOR = 4.48), and nonvaccination (weighted OR = 0.38)—mirror those in nonpregnant populations, where meta‐analyses show that severe acute disease and preexisting conditions (like asthma, OR = 2.15) are significant risk factors.^6^ This implies shared mechanisms, such as endothelial inflammation and viral persistence.^26^ However, pregnancy introduces unique nuances not captured in general population studies. Most notably, gestational timing emerged as an independent predictor, which may stem from trimester‐specific immune adaptations amplifying vulnerability early in gestation. Vaccination also modified symptoms (e.g., reduced neuropsychiatric issues), possibly via pregnancy‐specific immune interactions.

However, the landscape of SARS‐CoV‐2 continues to evolve. Recent surveillance data from CDC shows that the LP.8.1 variant has been increasing rapidly in 2025.^17^ Our study did not assess how the follow‐up care might play into the next stage of the pandemic nor does it give advice about when and how pregnant women should get vaccinated. Thus, the implications of variant shift for vaccine effectiveness against Long COVID remain unclear, underscoring the need for continued monitoring and updated research.

There are some key limitations to our study that the reader should consider when interpreting our findings. First, the study execution within a single tertiary care center potentially limits the direct generalizability of our results to broader community settings or healthcare systems with different patient demographics or resources. Furthermore, our study lacked a concurrently recruited nonpregnant control group. This restricts our ability to isolate aspects of Long COVID solely attributable to the pregnant state versus postviral sequelae in general. We acknowledge that the overlap between some Long COVID symptoms and common pregnancy‐related complaints represents a limitation of our study. Although most women were interviewed several months after delivery and clearly described these manifestations as new or persistent sequelae following acute infection—rather than typical pregnancy‐related conditions—we recognize that a certain degree of symptom overlap cannot be completely ruled out, as also noted in previous research on Long COVID during pregnancy.

There are also several potential limitations related to our methods. Our reliance on retrospective chart review combined with prospective telephone interviews carries an inherent risk of recall bias concerning the precise timing, nature, and duration of symptoms, as patient recollection can change over time. We also acknowledge a form of selection bias as our cohort focused exclusively on hospitalized individuals—likely experiencing more severe acute COVID‐19 than the general pregnant population. Consequently, the observed 27.0% prevalence of Long COVID might overestimate the burden across all pregnant individuals infected with SARS‐CoV‐2, including those with milder outpatient cases.

Finally, information on potentially significant confounding variables, such as detailed socioeconomic indicators, specific patterns of healthcare utilization, or occupational exposures, was not uniformly available. Similarly, while data on country of origin were collected, we lacked consistent details on length of residence or citizenship status. This heterogeneity prevented a reliable categorization of “immigrant status,” a variable known to correlate with vaccination uptake.

While statistical approaches like IPTW and E‐value calculations were employed to mitigate confounding, the possibility of residual bias from unmeasured factors persists. We also faced limitations in consistently capturing precise details about vaccination, including exact timing relative to infection or gestation, or specific vaccine types received, hindering analyses of optimal vaccine strategies. Similarly, the lack of individual‐level viral variant data restricts the applicability of our findings, collected across multiple pandemic waves, to the current or future variant landscape.

Additionally, calendar time represents a significant potential confounder unaccounted for in our models. Vaccination was unavailable pre‐2021, and the study spans multiple pandemic waves with variant shifts (e.g., Wild‐type to Omicron), which may differ in Long COVID propensity.^27^ We also lacked data on prior infections, limiting adjustment for hybrid immunity. Restricting analyses to vaccine‐available eras would underpower the study; thus, the vaccination effect (IPTW OR = 0.38) should be interpreted cautiously, as residual time‐dependent bias may persist despite IPTW and E‐value assessments.

## 5 CONCLUSION

Our study found that approximately one in four pregnant women with COVID‐19 developed Long COVID syndrome. Preexisting respiratory disease and severe illness during acute infection emerged as strong risk factors for Long COVID development. Moreover, we identified a significant temporal relationship between infection timing, early in pregnancy, and Long COVID risk. Vaccination was associated with a significantly lower risk of Long COVID and a modified symptom profile.

These findings, supported by emerging evidence from recent systematic reviews and meta‐analyses, highlight the importance of preventive strategies and careful monitoring of high‐risk pregnant women following COVID‐19 infection.

Future research should address several key questions: the interaction between vaccination, disease severity, and Long COVID development; variant‐specific effectiveness; value of preexisting comorbidities that can alter immune response. Prospective studies with larger cohorts and longer follow‐up are needed to further understand the natural history, pathophysiology, and optimal management of Long COVID in the obstetric population. Given the evolving nature of SARS‐CoV‐2, continued surveillance and updated analyses will be essential to inform clinical practice and public health policies.

## AUTHOR CONTRIBUTIONS

The clinical study was designed, conceptualized, and supervised by Anna Fichera. Eleonora Biancareddu collected data and wrote the original manuscript draft. Marco Bozzo conducted data analysis and created visualizations. Mirabella Ezenwa and Emma Paola Ongarini collected and analyzed data. Anna Fichera and Federico Prefumo were responsible for the interpretation of the data. All authors reviewed, edited, and approved the final version.

## CONFLICT OF INTEREST STATEMENT

The authors declare no conflicts of interest.

## ETHICS STATEMENT

This study has obtained ethical approval from the Regional Ethical Review Authority (Comitato Etico Lombardia 6; NP 6458) on January 14, 2025. The study was conducted according to principles of the declaration of Helsinki. Informed consent was obtained from all participants prior to their inclusion in the study.

## Supporting information


Table S1.

## Data Availability

The data that support the findings of this study are available from the corresponding author upon reasonable request.
