# Supplementary material for: Long COVID following SARS‐CoV‐2 infection during pregnancy: An observational study in a large Italian hospital during the COVID‐19 pandemic
Source: Acta Obstet Gynecol Scand. 2026 Feb 13;105(4):604–12. doi: 10.1111/aogs.70127 (PMC13140548; doi:10.1111/aogs.70127)
Supplement: Supplementary file 1 — Table S1. [file AOGS-105-604-s001.docx]

**Table S1.** Follow-up questionnaire.

Date: //________

1. PERSONAL DATA

- Date of birth: //________ (age: _____)
- Place of birth: _____________________
- Address: _________________________
- Telephone number: _________________
- Email address: _____________________

2. LIFESTYLE

- Smoking: [ ] Never, ≥ 10 years ago [ ], Former (<10 years) [ ], Yes n°____ cig./day
- Alcohol: [ ] No [ ] Yes

3. HOSPITALIZATION FOR COVID-19

- Reason for hospitalization: __________________________________________
- Duration: ________ days
- Symptoms at admission:____________________________________________
- Course of hospitalization (eventual transfer to ICU, complications, other):
- Instrumental exams:
  - Chest X-ray: [ ] No [ ] Yes
  - Chest CT scan: [ ] No [ ] Y

4. PAST MATERNAL MEDICAL HISTORY

- Gynecological pathology: [ ] No [ ] Yes, specify: _____________________________________________________________
- Surgical interventions: _____________________________________________________________
- Chronic diseases (chronic hypertension and/or cardiac disease, respiratory system diseases, endocrine diseases, autoimmune diseases, other):

_____________________________________________________________

- Therapies: _____________________________________________________________

5. OBSTETRIC HISTORY G____ P____ CS____ SA____ TOP/TA____ IUFD____ EP____

(Translator's Note: G = Gravida, P = Para, CS = Cesarean Section, SA = Spontaneous Abortion, TOP/TA = Termination of Pregnancy/Therapeutic Abortion, IUFD = Intrauterine Fetal Demise, EP = Ectopic Pregnancy. You may want to spell these out or use the English standard abbreviations like G_ P_ SAB_ TAB_ C/S_ IUD_ Ect_)

6. COURSE OF PREGNANCY

- Arterial hypertension, hypertensive disorders, pre-eclampsia: [ ] No [ ] Yes
- IUGR (Intrauterine Growth Restriction): [ ] No [ ] Yes, at ____ wk
- GDM (Gestational Diabetes Mellitus): [ ] No [ ] Yes, from ____ wk, therapy: _________
- Preterm birth/mPPT/pPROM (preterm premature rupture of membranes): [ ] No [ ] Yes, week ____
- Other complications: ___________________________________________

7. DELIVERY

- Date of delivery: //________
- Gestational age: ____ weeks
- Induction: [ ] No [ ] Yes, reason: ____________________________
- Mode of delivery: [ ] SVD (Spontaneous Vaginal Delivery) [ ] CS (Cesarean Section) [ ] OD (Operative Vaginal Delivery) Reason: __________________
- Postpartum hemorrhage: [ ] No [ ] Yes
- Other complications: [ ] No [ ] Yes ___________________
- Newborn:
  - Sex: __________
  - Weight: ________ g
  - Apgar: /
  - NICU (Neonatal Intensive Care Unit admission): [ ] No [ ] Yes

8. POSTPARTUM COURSE [ ] Physiological [ ] Pathological Specify any complications:

9. ANTI-COVID-19 VACCINATION

- Did you receive the anti-COVID-19 vaccine before hospitalization? [ ] Yes [ ] No
- If yes:
  - Number of doses: ____
  - Type of vaccine received: [ ] Moderna [ ] Pfizer [ ] AstraZeneca [ ] Other: __________
- Have you received further vaccine doses after hospitalization? [ ] No [ ] Yes, number of doses: ____

10. LONG COVID SYMPTOMS Did the symptoms of the acute infection last for more than 12 weeks or did new ones appear within this timeframe? [ ] No [ ] Yes

If yes, specify which of the following: [ ] Persistent fatigue [ ] Dyspnea (shortness of breath) [ ] Cognitive disorders (e.g., difficulty concentrating, memory loss) [ ] Joint pain [ ] Myalgia (muscle pain) [ ] Chest pain [ ] Alterations in taste [ ] Alterations in smell [ ] Insomnia [ ] Gastrointestinal disorders [ ] Tachycardia /heart problems [ ] Other symptoms (specify):_____________________

11. IMPACT ON QUALITY OF LIFE

- To what extent have Long COVID symptoms affected your quality of life?
- [ ] Not at all [ ] Slightly [ ] Moderately [ ] Severely [ ] Extremely

12. IMPACT ON FUTURE PREGNANCIES

- Has the experience of pregnancy/childbirth during the COVID-19 pandemic influenced your future choices regarding new pregnancies? [ ] No [ ] Yes [ ] I don't know
- If yes, in what way? (Select all relevant options) [ ] I have decided not to have any more children [ ] I have postponed trying for a new pregnancy [ ] I have accelerated trying for a new pregnancy [ ] I am more anxious/worried about future pregnancies [ ]

13. IN CASE OF SUBSEQUENT PREGNANCY

- Any complications _____________________________
